# Assessment of Neuromuscular and Psychological Function in People with Recurrent Neck Pain during a Period of Remission: Cross-Sectional and Longitudinal Analyses

**DOI:** 10.3390/jcm11072042

**Published:** 2022-04-06

**Authors:** Ahmed Alalawi, Valter Devecchi, Alessio Gallina, Alejandro Luque-Suarez, Deborah Falla

**Affiliations:** 1 Physical Therapy Department, College of Applied Medical Sciences, Umm Al-Qura University, Makkah 24382, Saudi Arabia; amalawi@uqu.edu.sa; 2 Centre of Precision Rehabilitation for Spinal Pain (CPR Spine), School of Sport, Exercise and Rehabilitation Sciences, University of Birmingham, Birmingham B15 2TT, UK; vxd823@student.bham.ac.uk (V.D.); a.gallina@bham.ac.uk (A.G.); 3 Department of Physiotherapy, Universidad de Malaga, 29016 Malaga, Spain; aluques@uma.es; 4 Instituto de la Investigacion Biomedica de Malaga (IBIMA), 29010 Malaga, Spain

**Keywords:** whiplash, chronic neck pain, recurrent neck pain, cervical kinematics, neuromuscular function

## Abstract

The aim of this study was to examine for the presence of differences in neuromuscular and psychological function in individuals with recurrent neck pain (RNP) or chronic neck pain (CNP) following a whiplash trauma compared to healthy controls. A secondary aim was to examine whether neuromuscular characteristics together with psychological features in people with RNP were predictive of future painful episodes. Multiple features were assessed including neck disability, kinesiophobia, quality of life, cervical kinematics, proprioception, activity of superficial neck flexor muscles, maximum neck flexion and extension strength, and perceived exertion during submaximal contractions. Overall, those with RNP (*n* = 22) and CNP (*n* = 8) presented with higher neck disability, greater kinesiophobia, lower quality of life, slower and irregular neck movements, and less neck strength compared to controls (*n* = 15). Prediction analysis in the RNP group revealed that a higher number of previous pain episodes within the last 12 months along with lower neck flexion strength were predictors of higher neck disability at a 6-month follow-up. This preliminary study shows that participants with RNP presented with some degree of altered neuromuscular features and poorer psychological function with respect to healthy controls and these features were similar to those with CNP. Neck flexor weakness was predictive of future neck disability.

## 1. Introduction

A whiplash injury commonly results in ongoing pain and disability, reduced work capacity, fatigue, restricted involvement in sports, depression, frustration, and anger [1,2,3,4]. The term ‘whiplash associated disorder’ (WAD) describes this multitude of clinical manifestations that commonly occur following the injury [5]. Over the last 30 years, the number of patients presenting to hospitals with traffic-related whiplash injuries has increased globally [6], placing a significant burden on health care and insurance systems [7,8,9]. Chronic neck pain (CNP) refers to persistent pain which lasts more than three months [10], while recurrent neck pain (RNP) refers to neck pain that has occurred frequently with complete pain-free periods in between. [11]. Both are common following the first episode of neck pain.

Altered neuromuscular function is a common feature in patients with acute and CNP including those that have sustained a whiplash injury [12,13,14]. These changes include disturbances in muscle strength, muscle behaviour and proprioception [15,16,17,18]. Additionally, restricted neck range of motion (RoM), as a static measure of movement, has been extensively documented for patients with neck pain of both traumatic and idiopathic origin [19,20]. Measures of dynamic motion such as slower [19,20,21,22], and irregular neck movement [20,21,22,23], have also been observed in people with CNP, and are associated with kinesiophobia [24]. Earlier work suggested that some measures of neuromuscular function may not always return to values seen in asymptomatic people even when pain resolves [25,26].

Several original studies and systematic reviews have aimed to identify prognostic factors associated with poor outcomes following a whiplash injury [27,28,29]. High-quality evidence has shown that higher pain and disability post-injury in the acute phase, are the most consistent at predicting longer-term pain and disability [30,31]. However, the predictive ability of wide range of neuromuscular adaptations has not been conducted previously. Additionally, there is very limited evidence examining the presence of neuromuscular adaptations in patients with RNP when they are pain free, i.e., in a period of remission. A recent systematic review [32], aiming to determine whether neuromuscular adaptations exist in people with recurrent spinal pain found very low level evidence to support muscle activity changes in people with recurrent low back pain, especially greater co-contraction, redistribution of muscle activity, and delayed postural control of deeper trunk muscles. Reduced range of motion of the lumbar spine was also found. Meaningful conclusions on people with RNP could not be drawn since only one study was identified [33]. In that particular study, thirty people with recurrent episodes of neck pain of non-traumatic origin were included and neck proprioception and performance on the craniocervical flexion test (i.e., the maximum pressure maintained for 10 s) were examined [33]. Both measures were able to differentiate between people with RNP and asymptomatic controls (areas under the curve of 0.69 and 0.73, respectively). However, it should be noted that the participants with RNP were not entirely asymptomatic as they presented with mild neck pain (mean scores on numerical rating scale 3.13 ± 2.01) and disability (mean scores on the Neck Disability Index 10.7 ± 5.12).

Currently there is very limited evidence on whether people with RNP who are in complete remission from their neck pain continue to display changes in neuromuscular function or psychological features such as high levels of kinesiophobia which may impact on neuromuscular function. Additionally, the predictive ability of these features in people with RNP has not been previously investigated in people who have sustained a whiplash injury. Yet this is highly relevant since the identification of physical and psychological factors that may increase the risk of developing future episodes of neck pain would provide more specific direction for appropriate treatment for the prevention of repeated episodes of pain [34,35].

The first objective of this study was to determine whether neuromuscular function and selected psychological variables are altered in people with RNP following a whiplash injury when tested during a period of remission compared to healthy people and whether these factors are comparable between people with RNP and CNP. We hypothesised that people with RNP in pain remission would present with altered neuromuscular and psychological function similar to those present in people with CNP. A secondary objective was to investigate the predictive ability of a variety of neuromuscular and psychological features for the development of new pain episodes over 12 months in those with RNP. We hypothesised that a combination of neuromuscular and psychological features could predict future ongoing neck pain episodes over the 12 months of assessment.

## 2. Materials and Methods

### 2.1. Study Design

A cross-sectional observational study, followed by a longitudinal analysis for those with RNP, was conducted and is reported according to the guidelines in the Strengthening the Reporting of Observational Studies in Epidemiology (STROBE) statement [36], with the STROBE checklist available in Supplementary Table S1. The study was approved by the Ethical Review Committee of the University of Birmingham, UK (ERN_19-0564) and was conducted in accordance with the Declaration of Helsinki.

### 2.2. Participants

**Figure 1:**
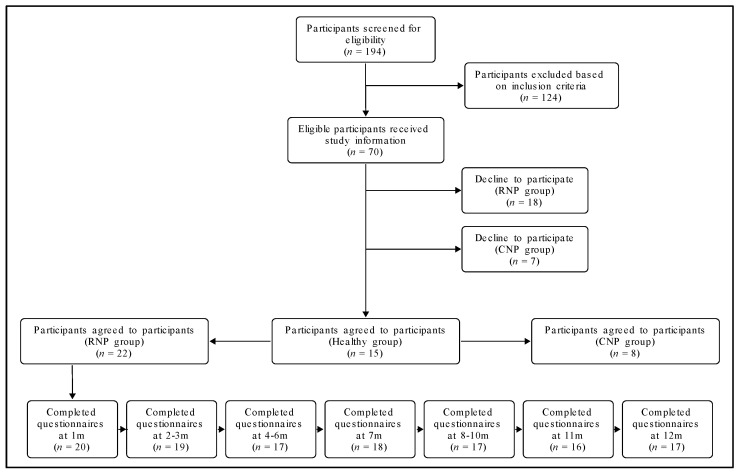
Flowchart of study population.

Three groups of adult participants (≥18 years old) were included in this study consisting of people with RNP, CNP, and healthy controls. A sample size of 15 healthy controls (mean age ± SD: 31.1 ± 5.7; female: 60%), 22 participants with RNP (mean age ± SD: 31.0 ± 11.8; female: 64%), and 8 participants with CNP (mean age ± SD: 33.6 ± 8.7; female: 88%) were included in this study (Figure 1). Those with RNP and CNP had a history of neck pain initiated following a whiplash injury, due to a motor vehicle collision. Further inclusion criteria for each group are described below.

#### 2.2.1. RNP Eligibility Criteria

Participants with RNP were included if they experienced two or more neck pain episodes (lasting ≥24 h) separated by a period of remission lasting at least 30 days during the previous 12 months, and experienced neck pain of at least 2/10 on the Numeric Rating Scale (NRS) [37] and lower than 10/50 on Neck Disability Index (NDI) [38] during an episode. These inclusion criteria are in line with the definition of recurrent low back pain [11]. Furthermore, individuals with RNP needed to be pain free at the time of assessment.

#### 2.2.2. CNP Eligibility Criteria

Participants in this group were included if their neck pain lasted three months or more, their current neck pain was at least 2/10 on the NRS, and they scored at least 10/50 on the NDI [38].

#### 2.2.3. Healthy Participants Eligibility Criteria

Healthy participants were required to have no current neck pain and no history of neck or shoulder pain that required treatment from a healthcare professional.

#### 2.2.4. Exclusion Criteria of All Groups

Participants were excluded if they participated in a neck or shoulder rehabilitation programme during the past three months or had any of the following: a history of neck or shoulder surgery [39], malignant spinal disorders, rheumatic condition, mental disorders [40,41], pregnancy, or regular use of analgesic medication prior to the injury due to chronic pain.

### 2.3. Recruitment

All participants were recruited from the community in Birmingham, UK, including staff and students at the University of Birmingham. The study was advertised using posters, local newspaper, and social media (Facebook) to expand the reach of the study. Initially, a researcher (AA) assessed the eligibility criteria of potential participants, sent the participant information sheet to participants via email, and answered any questions via email or telephone. Once an interested and eligible participant was identified, they were invited to attend one session at the University of Birmingham where the study was explained, a hard copy of the information sheet was provided, and written informed consent was obtained. Once consent was obtained, all participants were asked to complete self-reported questionnaires and undergo physical testing which occurred on the same day.

### 2.4. Baseline Measures (Candidate Predictors)

#### Patient-Reported Outcome Measures

The number of episodes referred to the number of pain episodes (over that last 12 months) that lasted more than 24 h with at least 30 days remission. The average pain intensity during an episode was assessed using the Visual Analogue Scale (VAS) [42], ranging from zero (no pain) to 100 (worst pain imaginable). The validity and reliability of the VAS have previously been established [43,44,45]. Neck pain duration was calculated in months and assessed only for the participants with CNP. Current pain intensity (for the CNP group only) was assessed using VAS immediately prior to physical data collection, by asking participants to indicate their current neck pain intensity.

To assess perceived neck disability at baseline, the NDI [38] was used which consists of 10 items related to daily activities such as reading, lifting, driving, personal care, work, sleeping, and recreation [38]. Each question has five ordinal response options from 0 (no disability) to 5 (complete disability) and the NDI scores are interpreted as recovered (NDI < 8), mild pain and disability (NDI 10–28), moderate/severe pain and disability (NDI > 30) [46]. The NDI is a valid and reliable measure in individuals with neck pain disorders [47].

The Tampa Scale of Kinesiophobia (TSK-11) [48] was used to assess fear of movement or injury during activities. It consists of 11-items producing a score which ranges from 11 to 44 with higher scores representing higher kinesiophobia. Scores greater than 37 are considered a high degree of kinesiophobia [49]. The reliability and validity of TSK-11 have been established [50].

Health-related quality of life was quantified using the European Quality of Life—Five Level (EQ-5D) scale that produces a single index value of range 0 to 1 where 1 is perfect health, and a VAS score ranging between 0 and 100, representing ‘worst’ to ‘best’ imaginable health state, respectively [51]. The EQ-5D, with each item having 5 possible responses, has improved inter-observer [ICC 2,1 0.57] and test-retest [ICC 2,1 0.69] reliability compared to the previous EQ-5D with three levels only [52]. The EQ-5D exhibits excellent psychometric characteristics across a wide variety of populations including musculoskeletal conditions [53].

Borg’s scale (6–20) [54] was used to assess participants perceived effort performing submaximal contractions of their neck muscles.

### 2.5. Testing Procedures

Initially, all participants completed baseline self-reported outcomes, prior to physical data collection (Table 1). All participants, including healthy controls, provided their demographics and completed measures of neck disability (NDI), kinesiophobia (TSK), and quality of life (EQ-5D). Further questionnaires related to previous pain episodes and duration of neck pain were completed by individuals in the RNP group, and CNP group, respectively.

#### 2.5.1. Cervical Kinematics

Physical testing was conducted by a physiotherapist in a quiet room. Each test was carried out with the participant seated in a chair with their arms supported and their feet on the ground. The assessor fixed an Inertial Measurement Unit (IMU; Noraxon USA Inc., Scottsdale, AZ, USA) on the middle of patient’s forehead and another over the thoracic spine (T1); the sensors were calibrated to zero with the head in a natural position. Participants were then instructed to perform active neck movements as far as possible, at a self-paced natural speed, since most daily activities are performed at a natural speed [22,55]. This approach is consistent with what has been described in previous studies [21,56].

The directions of the head movements were performed in the same order among participants. Firstly, active neck flexion/extension was performed by instructing the participant to look forward, then fully flex and extend their neck continuously over 10 cycles (repetitions) without stopping. The choice of 10 repetitions for neck movements was selected in accordance with previous studies involving people with neck pain [57] and healthy volunteers [58]. Furthermore, this reasonably large number of repetitions was necessary to produce a representative sample of natural head motions, yet without inducing dizziness [57].

Similar procedures were applied to the active rotation task, where participants performed 10 cycles of continuous right to left rotations. Participants were instructed to perform all movements at a pace that is similar to what they perceive as a normal speed. A short rest of 1 min was given between each movement direction, with a longer period provided if requested, although this was not required.

#### 2.5.2. Neck Proprioception

Once cervical kinematic examinations were completed, a rest of 3 min was provided, after which neck proprioception was assessed. Participants performed three repetitions of right and left neck rotation and in each trial, they were instructed to memorize a self-selected neutral position (starting position), close their eyes, and perform active head rotation after which they should return to the starting position as accurately as possible. All participants performed the proprioception test in the same order by alternating between right and left rotation with a rest period of one minute between each movement. The total testing time for the assessment of active neck movement and proprioception was approximately 15 min.

#### 2.5.3. Craniocervical Flexion

Tests of craniocervical flexion were performed involving two Maximum Voluntary Contractions (MVCs) of craniocervical flexion followed by four submaximal contractions (20%, 40%, 60%, 80, and 100% of MVC). To assess the MVC, craniocervical flexion strength testing was performed with the participant lying supine with the hip and knees flexed to approximately 90 degrees [13]. The head was placed in neutral position and a dynamometer (NOD; OT Bioelettronica, Turin, Italy) was placed behind the upper cervical region with the instruction “to nod as if saying yes but as hard as you can, without lifting the head off the bed”. Each maximum MVCs lasted 3 s, separated by 1 min rest in between repetitions [59].

In the same position described for the MVC, participants were instructed to perform craniocervical flexion at 20%, 40%, 60%, and 80% of their maximal force, attempting to hold the force for 10 s at each level. Visual feedback on force displayed on a tablet was used to guide the participant to reach and maintain the target force for the duration of the contraction. During this task, the amplitude of sternocleidomastoid (SCM) activity was measured with electromyography (EMG) (see details below).

#### 2.5.4. Maximal Neck Extension/Flexion (Isometric Contractions)

Two MVCs of both neck flexion and extension were performed using a Multi-Cervical Unit (MCU) (BTE Technologies Inc, Hanover, MD, USA); each MVC lasted 3 s with one minute rest in between. Participants were comfortably seated on the chair of the MCU with their hips and knees flexed to 90 degrees, their head in neutral position and feet flat on the MCU stand. To measure neck flexion strength, the load cell of the MCU was placed over the forehead and the participant was instructed to “push as hard as you can as you try to bring your chin to your chest” [18]. Once two trials were completed, the load cell was then placed on the back of the head and the patient was instructed to “push as hard as you can into the load cell as if trying to bring the back of the head to your neck” [18].

In the same positions described for the MVC, the participants were instructed to perform a single submaximal contraction at 20% of their maximal force and hold this for 10 s for both neck flexion and extension. During these tasks, the amplitude of both SCM and splenius capitis (SC) activity was recorded with EMG.

### 2.6. Instrumentation

#### 2.6.1. Inertial Measurement Unit

Neck kinematic and proprioception assessments were collected using a wearable IMU (Research PRO IMU, Noraxon USA Inc., Scottsdale, AZ, USA), with a sampling rate of 100 Hz. The dimensions of the sensor are 37.6 × 52 × 18.1 mm, and its mass is 34 g. The two sensors were fixed over the participants’ forehead and thoracic spine (T1) [22], using double-sided tape. The signal was acquired using the software myoRESEARCH 3.12 (Noraxon USA Inc., Scottsdale, AZ, USA).

#### 2.6.2. NOD Dynamometer and Multi-Cervical Unit (MCU)

Neck flexion and extension force was measured with the MCU (BTE Technologies Inc., Hanover, MD, USA). The reliability of measuring cervical strength with the MCU has been established (ICC ranging from 0.92 to 0.99) in individuals with neck pain [60]. Craniocervical flexion force was measured using a NOD device (OT Bioelettronica, Turin, Italy), a hand-held dynamometer.

#### 2.6.3. Electromyography Analysis

Surface EMG (Ultium^®^ EMG System, Noraxon USA Inc., Scottsdale, AZ, USA) was acquired from the SCM and SC bilaterally during the maximal and submaximal neck flexion and extension contractions whereas SCM only was measured during the submaximal craniocervical flexion contractions.

The skin was first shaved, if needed, rubbed with gel (Nuprep, Weaver and Company) and then washed with water using cotton wool. Noraxon dual EMG wet-gel electrodes (EMG electrodes, Noraxon USA Inc., Scottsdale, AZ, USA) were utilised which are disposable, wet-gel, self-adhesive Ag/AgCl snap electrodes. The electrode has an adhesive area of 40 mm × 22 mm, with dual circular electrodes of 10 mm diameter, and a fixed inter-electrode distance of 20 mm. Electrodes were placed “over the distal one-third of the muscle (sternal head)” [61] for the SCM muscle, and “at C2-C3 level between the uppermost parts of SCM and upper trapezius muscle” for the SC [62].

Raw data were collected via the Ultium EMG sensor (Noraxon USA Inc., Scottsdale, AZ, USA) using the Noraxon MyoMuscle software (myoRESEARCH, Noraxon USA Inc., Scottsdale, AZ, USA) which was then transferred to Matlab (Mathworks Matlab 2019b) for processing. EMG signals were low-pass filtered (pass band 20–400 Hz; order: 4) as used previously [63]. The EMG signals were sampled at 2000 Hz and converted with a 16-bit A/D converter.

### 2.7. Baseline Objective Measures (Candidate Predictors)

All data were analysed in Matlab (Mathworks Matlab 2019b). Signals related to neck movement were low-pass filtered (cut-off frequency of 10 Hz; order: 10) before computing the kinematic features. The start and end of the movement were defined as the time when the angular velocity exceeded a threshold of 5% of the peak velocity [22]. Although some studies used a threshold of 10% of the peak velocity to determine the start and stop of movement, using a threshold of 5% was deemed appropriate since we hypothesized that patients with RNP and CNP may present with lower peak velocity, therefore minimizing loss of data during the analysis. Moreover, the choice of 5% threshold was tested on our data during the pilot study of this project and considered appropriated for retaining representative data.

Maximum neck RoM (°) was defined as the maximum range achieved during each repetition of flexion, extension, and right and left rotation. The mean value of the ten repetitions for each direction was calculated and included in the analysis.

Mean velocity (Vmean [°/s]) was determined as the mean angular velocity achieved over the five repetitions for each movement direction. The average of the ten values was included in the analysis for each movement direction.

Peak velocity (Vpeak [°/s]) refers to the highest velocity value for each movement; the average of the ten repetitions were included in the analysis for each movement direction.

Number of velocity peaks (NVP [*n*]) refers to the number of times that the angular acceleration curve crossed zero. Details of this are reported elsewhere [64]. The average NVP that occurred across the ten repetitions were combined and included in the analysis for each movement direction.

Joint position error (JPE [°]) refers to the difference in degrees between the participants head position upon repositioning and the start location. The mean value of the three repetitions for each direction was calculated and included in the analysis.

Maximum craniocervical flexion strength (CCF MVC [Newton: N]) refers to the highest score achieved following the two maximal isometric contractions. Muscle activity during submaximal CCF contractions refers to the normalized EMG amplitude achieved during each of the four levels of submaximal isometric contractions (20%, 40%, 60%, and 80% of CCF MVC force). A 1 s sliding window was used to estimate the amplitude as a maximal root mean square (RMS) [65]. Two RMS values (for the right and left SCM) were obtained for each level of submaximal isometric contraction and these values were then normalized relative to the maximum EMG amplitude measured during the CCF MVC. The mean of both normalized values (right and left SCM) was included in the analysis [66].

Maximum neck strength in flexion and extension (MVC flexion and extension [kg]) refers to the peak force achieved following the two repetitions of each maximal neck isometric contractions.

Perceived exertion during the submaximal task in flexion and extension (Borg’s flexion and extension) refers to the value of perceived exertion assessed on Borg’s scale (6–20) [54] recorded immediately after completing the submaximal isometric contraction in flexion and extension at 25% MVC sustained for 30 s.

### 2.8. Outcome Measures for the Longitudinal Analysis (Prediction Model)

Two outcome measures were used to evaluate the predictive ability of physical and psychological measures (Table 1) in patients with RNP following a whiplash injury. All outcomes were treated as continuous variables without dichotomisation. This approach follows the recommendations of the PROGRESS series, that analysis of continuous variables be on a continuous scale [67]. This method increases the statistical power and reduces information loss.

To collect the outcome measures in this study, for each month of a 12-month follow-up, participants were instructed to record their neck disability, number of days with neck pain, and the average pain intensity during the previous month. These data were recorded each month using the electronic system Research Electronic Data Capture (REDCap) which enables researchers to monitor and manage the data collection process via a web interface [68]. The system provided an individualised link, involving the outcome measures, that was sent automatically each month for each participant.

#### 2.8.1. Primary Outcome

The NDI score was selected as the primary outcome, which was assessed six months following baseline assessments. Using six months as a cut-off for identifying outcome was selected a priori [69,70]. NDI is widely used to evaluate perceived neck disability in people who have sustained a whiplash injury [71,72], and is a reliable and valid outcome [47].

#### 2.8.2. Secondary Outcome

The secondary outcome was the number of days with pain. The mean number of days with pain over the course of 12 months considered. This outcome was defined as the number of days with neck pain during the previous month that lasted at least 24 h, with pain intensity of at least 20/100 on a VAS. This was measured using the questions ‘Over the past month, how many days have you experienced neck pain?’ and ‘Over the past month, how would you rate your average neck pain intensity?’. The response for the first question is an absolute number, while a VAS score (0–100) was used to quantify pain intensity. The outcome and its definition have been used before by participants with low back pain [73], although pain intensity was assessed on a scale from 0–10. The selection of this outcome is of clinical importance as it captures pain that is relevant to the patients [74]. The mean number of days with pain per participant across the 12-month follow-up period was included in the analysis.

### 2.9. Sample Size

A sample size of 50 participants with RNP, 15 with CNP, and 15 healthy controls was initially planned. These numbers were not achieved, except for the control group, due to the COVID-19 pandemic which severely disrupted data collection for this project. Nevertheless, the current sample size is comparable to similar research that examined the same spine kinematic and neuromuscular characteristics in patients with neck [22] and/or low back pain [75].

### 2.10. Statistical Analyses

#### 2.10.1. Cross-Sectional Analysis

Descriptive statistics were performed for participant demographics, and the data from self-reported questionnaires, cervical kinematic features, proprioception, and maximal and submaximal tasks. The normality of data distribution for self-reported and objective measures was assessed using the Shapiro–Wilk test. If data were not normally distributed for the measure of interest (*p* ≤ 0.05), differences among groups were assessed using the Kruskal–Wallis test, after which a post hoc test (Dunn’s test) was performed for making multiple pairwise comparisons.

If data were normally distributed (*p* ≥ 0.05) for a measure, the following steps were conducted. Initially, homogeneity of variance was assessed using Levene’s test for equality of variances. If a feature was homogenous (Levene’s test value: *p* ≥ 0.05), results from one-way analysis of variance (ANOVA) were used. When a feature was non-homogenous (Levene’s test value: *p* ≤ 0.05), results from a Welch ANOVA were used. Finally, a Tukey post hoc test was performed following one-way ANOVA, while Games–Howell post hoc test was used following Welch ANOVA. 

#### 2.10.2. Longitudinal Analysis

To identify the predictive value of baseline measurements on NDI at 6 months and on future episodes with neck pain over 12 months period, a two-step modelling approach was used [76]. Firstly, least absolute shrinkage and selection operator (LASSO) regression was used to reduce the number of candidate predictors entering into second stage analysis. A fivefold cross-validation was used in this study, considering the sample size. Further details about LASSO regression [69,77,78] and cross-validation [79] have been reported elsewhere. LASSO regression was used in the current study as it is feasible for estimating models with multiple predictors in a small sample size [80] and avoiding overfitting the data [81]. The analysis was performed on all baseline candidate predictors reported in Table 1. Candidate predictors with no predictive power or those that were highly correlated were penalized and reduced to zero. This penalisation (shrinkage) approach is used to effectively exclude candidate predictors from the final model by shrinking their coefficients to exactly zero [77]. Candidate predictors with zero coefficients were excluded from entering stage two. The second step was to perform multivariate linear regression analysis on candidate predictors with regression coefficients of more than zero that were identified from LASSO (first stage). R statistical software was used to conduct this analysis. The functions, packages, and codes that were used to analyse this data have been described elsewhere [82].

For this study, data from individuals with full cases for each model were considered. As a result, the observation number differs between models. This approach was used previously in [83]. For example, 17 participants with complete data were considered to develop the model with NDI, while 19 were considered for the model involving the outcome of number of days with pain.

Multiple imputations to deal with missing data in this study were not used. This is because all missing data were in the dependent variables (outcomes). Moreover, according to a previous study, multiple imputation is unnecessary for analysing longitudinal data as findings showed that multiple imputation was highly unstable when the multiple imputations were repeated 100 times [84].

The mean squared error (RMSE) [85] was used to quantify the prognosis error between predicted and observed values in each generated prognostic model. This is a measure to assess the internal validity of a model [86]. RMSE is interpreted on the same scale of an outcome. For example, NDI scores range from 0 to 50, and therefore RMSE can range from 0 to 50 too.

## 3. Results

### 3.1. Characteristics of Participants

Demographic characteristics and results for the self-reported questionnaires at baseline are reported in Table 2, with further figures available in the Supplementary Materials. Mean age (SD) was 31.1 ± 5.0 for the healthy participants, 31 ± 11.8 for RNP, and 33.6 ± 8.7 for those with CNP; the majority were females in all three groups. No significant differences were observed in participant demographics, except for height (*p* = 0.02). The mean score of average neck pain intensity for those with RNP during an episode (56.4 ± 14.5) and those with CNP (56.1 ± 19.5) was similar.

Descriptive statistics of the self-reported questionnaire measured at baseline for the three groups are provided in Table 2. Neck disability measured by the NDI (χ^2^ (2) = 32.34, *p* < 0.0001) and quality of life by EQ-5D (χ^2^ (2) = 23.03, *p* < 0.0001) were significantly different across all three groups. Patients with CNP presented with the highest disability (17.5 ± 7.6), followed by RNP (5.5 ± 3.2), and healthy controls who had almost no disability as expected (0.7 ± 1.1). The opposite was observed for quality of life where participants with RNP (0.92 ± 0.09), and CNP (0.68 ± 0.21) had significantly lower scores compared to healthy controls (0.98 ± 0.04), indicating lower quality of life. The Tukey post hoc comparison test revealed significant differences in TSK between those with RNP and healthy controls (*p* < 0.001), and between CNP and healthy controls (*p* < 0.0001), but not between RNP and CNP (Table 2). Significant differences were observed for EQ-VAS between RNP and CNP (*p* < 0.05), and between healthy controls and CNP (*p* < 0.001).

### 3.2. Cervical Kinematics and Proprioception

The descriptive statistics and the results of the one-way ANOVA for cervical kinematics and proprioception are reported in Table 3. People with RNP showed no significant differences when compared to healthy or CNP groups in RoM, but significant differences were observed between CNP and controls in combined RoM in flexion and extension (*p* < 0.05), and combined right and left rotation (*p* < 0.05). JPE following right (χ^2^(2) = 0.08, *p* = 0.96) and left (χ^2^(2) = 0.58, *p* = 0.75) rotations were not significantly different among groups. Mean velocity was significantly lower in those with RNP and CNP than healthy controls during neck flexion (χ^2^(2) = 12.98, *p* = 0.0015) right rotation (*F*(2,39) = 5.24, *p* = 0.01), and left rotation (*F*(2,39) = 5.53, *p* = 0.008), but not during neck extension (χ^2^(2) = 4.81, *p* = 0.09). Neither group with neck pain showed significant differences in mean velocity during any movement direction.

The NVP were higher (less smooth movement) in all directions in those with RNP and CNP compared to healthy controls. However, significant differences for the RNP group were only observed during flexion and left rotation (*p* < 0.05), and during both rotations for those with CNP (*p* < 0.05). Both groups with neck pain showed similar NVP with no significant difference between groups.

### 3.3. EMG Amplitude Assessed during Submaximal CCF Contractions

Maximum CCF strength did not differ across groups (*p* = 0.57). The activity of SCM during the submaximal CCF contractions at 60% MVC was significantly different between people with CNP and RNP (*p* < 0.01) and between CNP and healthy controls (*p* < 0.01). No other significant differences were found. Data are summarised in Table 4.

### 3.4. Maximal Neck Strength and Perceived Fatigue

A significant difference was observed between people with RNP and controls for neck extension strength (*p* < 0.05), but with no significant difference between RNP and CNP groups. No difference in neck flexion strength was observed between groups. People with RNP and CNP displayed similar greater perceived exertion in flexion and extension. Perceived exertion assessed during the submaximal isometric neck flexion was significantly different between those with RNP and controls (*p* < 0.01). Results are summarised in Table 5.

### 3.5. Participant Follow-Up through the Longitudinal Analysis

The total numbers of participants who completed the follow-up questionnaires at each month are reported in Figure 1. From 22 participants who participated at baseline, 17 (77%) participants completed the NDI at six months, whereas 19 (86%) completed the outcomes related to number of days with pain.

Two participants did not complete any of the 12-month follow-up questionnaires despite the maximum of three reminders. The highest completion rate of follow-up was at the first month (*n* = 20; 91%), whereas the lowest was at 12 months (*n* = 16; 73%). One participant withdrew from the study at three months without providing any reason. No significant differences in baseline characteristics were present between the participants who dropped out and those included in the current study.

#### 3.5.1. Characteristics of Participants

Self-reported outcomes indicated that, on average over the 12 months, people complained of neck pain for an average of five days per month. The mean of monthly number of days with pain for all participants is illustrated in Figure S8. Mean neck disability assessed by the NDI was (mean ± SD) of 8.6 ± 5.0 at six months.

#### 3.5.2. Step 1: Predictor Variable Selection (i.e., Shrinking the Number of Predictors)

The baseline covariates for both outcomes (NDI and future episodes of neck pain) that had nonzero coefficients are reported in Table 6. Using LASSO, the number of predictors for the outcome NDI at six months was reduced from fifteen to two predictors including MVC in flexion and previous number of days with pain. For predicting the outcome future episodes of neck pain at one year, the number of predictors was reduced from fifteen to one which was previous number of pain episodes. These variables for the two outcomes were included in the multivariate regression analysis in the next step. Graphs of the reduction in number of predictors achieved by applying LASSO are available in the Supplementary Materials (Figures S9 and S10).

#### 3.5.3. Step 2: Prediction Model Development

##### Prediction of Neck Pain and Disability at Six Months

**Figure 2:**
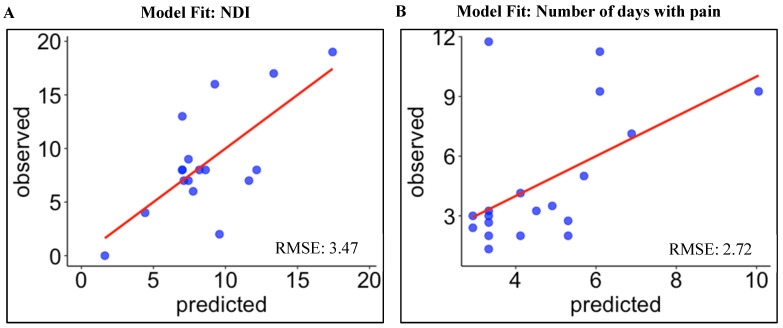
Scatterplots of two models fit comparing the predicted and observed values for each outcome: NDI: Neck Disability Index at six months (**A**) and number of days with pain over the 12-month follow-up period (**B**). The diagonal line in red indicates perfect prediction. RMSE: root mean square error, which represents the error between predicted and observed values in each generated prognostic model. Lower values of RMSE indicate better prediction.

A multiple regression was run to predict NDI at six months from MVC during flexion and previous number of neck pain episodes. These variables significantly predicted the NDI at six months, *F*(2,14) = 6.97, *p* = 0.008, R^2^ = 0.50. Both variables added significantly to the prediction model and are reported in Table 7. A one-kg reduction in MVC in flexion significantly increased NDI by 0.32 units (*t* = −2.21, *p* = 0.04, 95% CI: [−0.64]–[−0.01]). A single episode of neck pain within the last 12 months significantly predicted an increase in NDI by 0.54 units (*t* = 2.56, *p* = 0.02, 95% CI: 0.09–0.99). This model explained 43% of the variability in NDI at six months. This model resulted in a RMSE of 3.47 meaning that the NDI values that were predicted by this model differed from the observed values of NDI by 3.47 (Figure 2A).

##### Prediction of Future Episodes of Neck Pain over the 12-Month Follow-Up Period

A multiple regression was run to predict future episodes of neck pain within the next year, from previous number of pain episodes. This variable resulted in a statistically significant model predicting future episodes of neck pain, *F*(1,17) = 6.93, *p* = 0.017, R^2^ = 0.29. A single episode of neck pain within the last 12 months significantly predicted a future episode by 0.40 unit (*t* = 2.63, *p* = 0.02, 95% CI: 0.08–0.71) (Table 8). This model explained 25% of the variability in future episodes of neck pain. The RMSE for this model was 2.72, representing the differences in number of days between the predicted and observed values (Figure 2B).

## 4. Discussion

This is the first study to conduct a comprehensive investigation of neuromuscular features including cervical kinematics, sensorimotor performance (proprioception), superficial neck muscle activity, neck strength, and subjective fatigue among individuals with CNP, RNP (following a whiplash injury), and healthy controls. The findings provide evidence that people with a history of neck pain, even when in remission from pain, present with similar psychological and neuromuscular function consisting of altered neck movement, increased activity of superficial neck muscles, lower neck muscle strength, and greater perceived fatigue during sustained contractions. Importantly, when examining the predictive capacity of these features, lower neck flexion strength together with a higher number of previous pain episodes within the last 12 months were predictors of higher neck disability at six months. This provides preliminary evidence that some aspects of neuromuscular function (namely lower neck strength) are relevant for predicting future neck pain and disability.

The current study showed that people with either CNP or RNP following a whiplash injury presented with higher disability, higher kinesiophobia, and lower quality of life compared to healthy controls. The presence of psychological features and poorer quality of life have been commonly reported previously for patients with chronic WAD [87]; however, this is the first study to demonstrate that people with frequent episodes of neck pain could present with disability, poorer quality of life and some degree of kinesiophobia despite being pain free.

### 4.1. Cervical RoM

A general trend of reduced RoM was observed for both the CNP and RNP groups although significant differences were only observed between CNP and controls. Reduced RoM either in all or some directions has been reported previously in patients with CNP [19], despite methodological differences between studies. Whilst not significant, the average cervical RoM was lower in people with RNP compared to the controls. This might be due to the small sample size in the current study, which could result in this study being underpowered for RoM. The extent of restricted cervical RoM in people with RNP has not been studied before [32], but restricted RoM in the thoracic and lumber spine was reported in people with recurrent low back pain [88,89,90]. However, unlike the current study, the studies on recurrent low back pain included participants that reported some degree of pain during the assessment [88,90]. Future research should further investigate the presence of changes in spine kinematics in people with RNP [32] in a larger sample size.

### 4.2. Velocity and Smoothness of Neck Movement

Individuals with CNP in the current study moved their neck slowly and with irregular movements when performing cervical rotations. These findings are similar to previous work showing that people with CNP, either from traumatic or non-traumatic causes, display more irregular and slower neck movement [19,20,21,22]. Such a pattern of movement could be interpreted as cautious movements to avoid neck pain [91]. These changes in how neck movements are performed are in line with current theories regarding how pain affects movement and motor control [91]. However, the current study uniquely showed that slower neck movement in flexion and rotation with irregular neck movements in flexion and left rotation can also be present even when pain is not present, i.e., during a period of remission in people with RNP. The driving mechanism for the altered movement performance (slow and irregular movement) during pain remission is not fully understood and further studies exploring these neuromuscular adaptations and their association to clinical features should be investigated.

### 4.3. Cervical Proprioception

In this study, neck proprioception was not significantly different between groups. This finding was also observed in previous studies of patients with persistent WAD, who have similar pain intensity to the cohort tested in the current study [92,93,94]. A recent meta-analysis, found that patients with chronic WAD have significant larger JPE following cervical rotation when compared to healthy controls, but there is a discrepancy between studies [16]. Such discrepancies could be attributed to various factors. For example, several studies have used different methods to assess JPE including a variety of measurement devices and sensor placements [95] that potentially influenced the findings. Moreover, people with chronic WAD presenting with dizziness or greater pain intensity tend to show greater deficits in sensorimotor control [96], and this was not accounted for in the current study. Finally, sensorimotor disturbances are highly variable between people with WAD in both the nature of impairments and their frequency of presentation [96] and thus our sample size may have not been sufficient to capture a difference.

### 4.4. EMG Amplitude Assessed during CCF Submaximal Contractions

The current study showed generally higher activity of the SCM in people with CNP compared to healthy controls, although significant differences were only seen at 60% MVC. Once again, the small sample size could be the reason for why this was significant at 60% only and not at other levels. Previous studies showed that people with CNP often display higher activation of the superficial neck flexors [13,15,97,98], which is negatively associated with the extent of activation of the deep neck flexors [66]. The effect of pain on coordination between the deep and superficial neck flexors is well documented [99,100,101], and such a phenomenon was also seen early in patients with acute neck pain following a whiplash trauma [17]. Notably, greater activation of the superficial neck muscles was generally seen in this study (albeit not significant) even during remission of pain in people with RNP following a whiplash injury. It could be hypothesised that there might be ongoing motor control deficits for these individuals which have not been specifically targeted during a period of rehabilitation. For example, studies have shown that neuromuscular dysfunction can persist despite the resolution of, or reduction in, pain following active interventions not specifically designed to alter neuromuscular control [102,103].

### 4.5. Maximal Neck Strength and Perceived Fatigue

Both groups with a history of neck pain displayed lower isometric neck flexion and extension strength, although significant differences were only observed in extension between people with RNP and controls. People with neck pain frequently present with lower neck strength [18,104,105,106], though the degree of impairment varies greatly between patients [107] and can be associated with features such as the degree of kinesiophobia [59] and current pain intensity [108]. Previous work has shown that, compared to healthy controls, individuals with persistent WAD have significantly lower isometric MVC force in extension, retraction, and lateral flexion [18]. However, the current study was not able to confirm these findings. These differences could be explained due to the natural variability in neck strength among participants [91]. A large range of neck strength values has beenshown previously in people with CNP, most likely reflecting the large heterogeneity observed among people with neck pain [18,109,110,111]. Another reason could relate to the level of disability, since strength deficits are typically larger in those with higher disability [18].

Besides lower neck strength, higher perceived fatigue during neck flexion (significantly different) was found in the group with RNP during the submaximal contraction at 25% MVC. Previous studies found evidence of greater neck extensor endurance than neck flexor endurance in people with idiopathic neck pain [112,113,114], which could explain why significant differences were observed in flexion only. Indeed, the CNP and RNP groups had a mean score of approximately 15 on Borg’s scale in flexion compared to a mean of 10 for extension.

### 4.6. Predicting Neck Disability and Number of Days with Pain

In our sample, higher number of pain episodes within the last 12 months was a common predictor of higher neck disability and a higher number of days with pain. This finding is consistent with a previous prognostic study of people with RNP who were followed for one year [115]. The study found that a previous episode of neck pain predicted future recurrence of pain, which was defined as a new episode of neck pain [11]. Another study in people with low back pain confirmed the negative effect of a longer duration of a current episode on disability up to five years [116]. Nonetheless, no study has investigated this in people with RNP following a whiplash trauma, which warrants further investigation.

Besides the higher number of pain episodes, baseline lower isometric neck strength in flexion was identified as a predictive factor of higher disability at six months. Although not directly comparable to the current study, previous studies found similar findings in that muscle strength was a significant factor predicting future injury in the lower limb [117,118,119,120]. Lower neck strength in flexion was observed at baseline in patients with RNP, who presented on average with a reduction (−5.6 kg) in neck strength in flexion compared to healthy controls. These findings could emphasize the potential long-term effect of impaired neck strength and frequent episodes of neck pain on the development of neck disability. Further studies are needed to confirm this finding and investigate the interaction between neck muscle strength and future episodes of neck pain.

### 4.7. Model Performance

In this study, our models performed similarly to earlier machine learning prediction models. The first model in this study provided an estimate of the expected NDI values at six months with an average RMSE of 3.47 points, on a 0–50 scale. This score represents the average magnitude (error) of the difference between the observed NDI at six months and scores predicted by the model. In another words, it measures how close the observed data points are to the predicted model values where lower RMSE values reflect a better fit.

The RMSE score to predict NDI is similar to a model generated in people with cervical radiculopathy [82], with an RMSE of about 8.2% (NDI 0–100% scale). However, this comparison should be interpreted with caution due to the different populations. The other developed model in the current study showed that the average difference between predicted and observed values, indicated by RMSE, was 2.72 days with pain.

### 4.8. Clinical Implications

The current study provided evidence that people with RNP presented with changes in some neuromuscular and psychological features even during complete remission of pain. Furthermore, some of these changes were comparable to people with CNP. These findings could have significant implications for rehabilitation and prevention. For example, some of the features could be targeted in a rehabilitation program with the aim to promote restoration of altered function identified in this study and preventing recurrent episodes of neck pain. Commonly treatment is aimed at reducing pain, yet this work emphasises that restoration of neuromuscular function is equally relevant.

The longitudinal investigation in the current study showed that a higher number of previous pain episodes together with lower neck flexion strength predicted higher neck disability six months later. Neck strength is a modifiable feature. Thus, strengthening of the neck flexors in people with RNP may lower future neck disability although this needs to be tested in a longitudinal study. On the other hand, although the number of previous pain episodes is not a modifiable variable, this should be considered.

### 4.9. Strength and Limitations

This study has several strengths. This is the first study to examine physical features in a group of participants with RNP following a whiplash injury who were asymptomatic at inception. Moreover, a comprehensive battery of measures including demographic, psychological, and physical features were assessed at baseline. All these baseline features were then included as predictors of outcomes in people with RNP who were followed up over 12 months. A follow-up rate of more than 80% is desired in prognostic research [121]. This cut-off was fulfilled in one of the developing models including 86% follow-up rate across 12 months study period. For prognostic analysis, best practice recommendations were followed for the development and validation of the models [122,123].

There are some limitations to consider. One of the main limitations of this study is the sample size which could bias the results of this study. A sample size of 50 participants for RNP, 15 for CNP, and 15 for controls was planned in advance, but this was fulfilled only in the latter group. This was because of the COVID-19 pandemic which interrupted data collection. However, the current study was able to find some significant differences across groups and/or show a trend at baseline. Another potential limitation is that the number of female participants was higher than males in the group with CNP. However, no significant differences were observed in gender across groups as reported in Table 2. For prognostic analysis, a low sample size in the RNP group prevents us from separating the data into training and validation sets, the latter could be used in independent validation [123]. Furthermore, this smaller sample size compared to the high number of predictors could lead to overfitting of the developed models. However, this study incorporated LASSO, a powerful method that performs regularization and feature selection and can deal with a high number of predictors [124]. This is a unique study and it is difficult to determine the extent to which the results are generalizable, especially given that a convenience sample was adopted. Additionally, this study may not be generalizable to people with greater neck pain and disability as this was associated with general variability of neuromuscular adaptations [15,125,126]. This study included people with RNP and CNP who experienced minimal and mild to moderate pain and disability [46], respectively. Similarly, the higher level of kinesiophobia in people with CNP in the current study may not be generalizable to other cohorts with CNP who present lower levels of kinesiophobia. Restriction in the range and performance of neck movements could be influenced by kinesiophobia [127,128]. As a further consideration, it should be noted that kinesiophobia was the only measure of psychological function that was assessed in the current study and other features such as anxiety and depression may be relevant.

## 5. Conclusions

Participants with RNP during a period of remission presented with altered neuromuscular function and poorer psychological function, and several of these features were comparable to the presentation of people with CNP. These features included higher disability, higher kinesiophobia, and lower quality of life. People with RNP also performed slower and more irregular neck movements in most directions and displayed lower neck strength in extension and higher perceived fatigue in flexion. Some of these baseline variables were able to predict ongoing neck disability and days with pain in those with RNP when followed over 12 months. These included a higher number of previous pain episodes and lower neck flexion strength.

## Figures and Tables

**Table 1:** Summary of collected data across groups and their time points.

Data Collection Point	Domain	Variables	RNP	CNP	Healthy Controls
Baseline	Demographics	Age	✓	✓	✓
		Gender	✓	✓	✓
		Height	✓	✓	✓
		Weight	✓	✓	✓
	Patient-reported measures	NDI	✓	✓	✓
		TSK-11	✓	✓	✓
		EQ-5D	✓	✓	✓
	Others	Number of neck pain episodes	✓		
		Average of pain episodes (VAS)	✓		
		Neck pain duration		✓	
		Current pain intensity		✓	
	Objective measures	Cervical kinematics (RoM, velocity, and smoothness)	✓	✓	✓
		Neck proprioception	✓	✓	✓
		Peak score of craniocervical flexion test	✓	✓	✓
		Muscle activity during submaximal CCF contractions	✓	✓	✓
		Maximum neck strength in flexion and extension (MVC flexion and extension [kg])	✓	✓	✓
		Perceived exertion during the submaximal task in flexion and extension (Borg’s scale)	✓	✓	✓
Outcome measures	Questionnaires	Number of days with pain	✓		
		Neck Disability Index (NDI)	✓		

RNP: recurrent neck pain; CNP: chronic neck pain; ✓: collected data.

**Table 2:** Baseline characteristics of all three groups.

	Groups			*p*-Value
	Healthy Control (*n* = 15)	RNP (*n* = 22)	CNP (*n* = 8)	
	Mean ± SD	Mean ± SD	Mean ± SD	
Age (years)	31.1 ± 5.7	31.0 ± 11.8	33.6 ± 8.7	0.24 ^1^
Gender (male:female (%))	6:9 (60%)	8:14 (64%)	1:7 (88%)	0.38 ^2^
Height (m)	1.7 ± 0.1	1.7 ± 0.1	1.6 ± 0.1	0.02 ^3^
Weight (kg)	69.1 ± 14.8	74.7 ± 18.0	59.5 ± 9.8	0.07 ^1^
NDI (0–50)	0.7 ± 1.1	5.5 ± 3.2 *	17.5 ± 7.6 *^,†^	<0.001 ^1^
TSK (17–68)	29.1 ± 4.3	35.2 ± 5.5 *	40.5 ± 7.5 *	<0.001 ^3^
EQ-5D (0–1)	0.98 ± 0.04	0.92 ± 0.09 *	0.68 ± 0.21 *^,^^†^	<0.001 ^1^
EQ VAS (0–100)	85.5 ± 10.2	78.5 ± 15.4	64.1 ± 14.4 *^,^^†^	0.005 ^1^
Number of pain episodes, 12 m	-	5.9 ± 4.4	-	
Average of pain episodes, VAS (0–100)	-	56.4 ± 14.5	-	
Current neck pain, VAS (0–100)	-	-	56.1 ± 19.5	
Neck pain duration, m	-	-	39.1 ± 41.4	

SD: standard deviation; NDI: Neck Disability Index; TSK: Tampa Scale of Kinesiophobia; EQ-5D: European Quality of Life—5 Dimensions; EQ-VAS; self-rated health on a vertical visual analogue scale; VAS: Visual Analogue Scale. ^1^ Kruskal–Wallis Test. ^2^ Chi-square Test. ^3^ One-way ANOVA (Bonferroni post hoc shows significant group difference in height between healthy and CNP [*p* < 0.02], and RNP and CNP [*p* < 0.03]). * Post hoc significant difference from control group at *p* < 0.05. ^†^ Post hoc significant difference from RNP group at *p* < 0.05.

**Table 3:** Summary statistics for the kinematic and proprioception features of all three groups with differences assessed using One-way ANOVA.

	Groups			*p*-Value
	Healthy Control (*n* = 15)	RNP (*n* = 22)	CNP (*n* = 8)	
	Mean ± SD	Mean ± SD	Mean ± SD	
Flexion				
Vmean (°/s)	72.8 ± 12.3	55.0 ± 18.5 *	42.9 ± 14.3 *	0.002 ^1^
Vpeak (°/s)	149.5 ± 33.9	114.0 ± 41.3 *	90.8 ± 28.8 *	0.004
NVP (*n*)	9.4 ± 4.0	17.1 ± 9.4 *	17.5 ± 8.2	0.005 ^2^
Extension				
Vmean (°/s)	66.5 ± 15.7	55.4 ± 21.2	46.7 ± 16.5	0.09 ^1^
Vpeak (°/s)	133.8 ± 31.5	111.0 ± 45.1	97.2 ± 34.4	0.12
NVP (*n*)	8.3 ± 4.1	17.8 ± 14.0	16.5 ± 9.0	0.066 ^1^
Right Rotation				
Vmean (°/s)	132.5 ± 29.3	101.5 ± 41.7 *	82.5 ± 22.0 *	0.001 ^2^
Vpeak (°/s)	244.7 ± 52.5	190.5 ± 76.7	157.1 ± 37.9 *	0.001 ^2^
NVP (*n*)	5.1 ± 3.3	8.6 ± 9.1	10.2 ± 6.5	0.017 ^1^
JPE	3.8 ± 2.1	4.4 ± 2.5	5.5 ± 5.9 *	0.76 ^1^
Left Rotation				
Vmean (°/s)	131.2 ± 30.7	100.1 ± 41.0 *	79.5 ± 22.6 *	0.001 ^2^
Vpeak (°/s)	244.5 ± 57.2	188.8 ± 71.7 *	148.7 ± 34.7 *	<0.001 ^2^
NVP (*n*)	3.7 ± 2.8	9.0 ± 8.8	11.6 ± 10.5	0.014 ^1^
JPE	4.2 ± 2.8	4.7 ± 2.8 *	5.2 ± 5.2 *	0.711 ^1^
Combined RoM				
Flexion/Extension	52.6 ± 8.1	49.5 ± 7.9	42.9 ± 10.2 *	0.041
Right/Left Rotations	71.5 ± 6.2	67.1 ± 9.4	62.1 ± 9.1 *	0.042

SD: standard deviation; SD error: Standard error (of the mean); CI: confidence intervals; RoM: Range of motion; Vmean: mean velocity; Vpeak: peak velocity; Vpeaks: mean of peaks velocity; NVP: number of velocity peaks; JPE: joint position error. ^1^ Differences were assessed using Kruskal–Wallis ANOVA. ^2^ Differences were assessed using Welch’s ANOVA. * Post hoc significant difference from control group at *p* < 0.05.

**Table 4:** Normalized EMG amplitude (%) recorded from sternocleidomastoid muscles during each of the five submaximal craniocervical flexion contractions in addition to the maximum craniocervical contraction.

	Groups			*p*-Value
	Healthy Control (*n* = 15)	RNP (*n* = 22)	CNP (*n* = 8)	
	Mean ± SD	Mean ± SD	Mean ± SD	
Normalized EMG amplitude (%)				
20%	18.8 ± 12.0	33.6 ± 22.6	52.0 ± 53.1	0.11 ^1^
40%	35.2 ± 23.9	64.3 ± 88.5	70.8 ± 36.5	0.07 ^1^
60%	50.9 ± 15.9	58.7 ± 29.0	111.8 ± 80.1 *^,†^	0.003
80%	66.9 ± 21.7	79.0 ± 33.6	108.6 ± 88.4	0.34 ^1^
Maximum craniocervical contraction				
CCF MVC (N)	52.1 ± 22.3	44.0 ± 23.4	47.1 ± 22.8	0.57

SD: standard deviation; SD error: standard error (of the mean); CI: confidence intervals, CCF MVC: maximum craniocervical flexion strength; N: Newton (unit of force). Numbers are presented as normalized EMG (%). ^1^ Kruskal–Wallis ANOVA. * Post hoc significant difference from control group at *p* < 0.05. ^†^ Post hoc significant difference from RNP group at *p* < 0.05.

**Table 5:** Results of neck strength during the isometric contraction and perceived fatigue during submaximal contraction in MCU.

	Groups			*p*-Value
	Healthy Control (*n* = 15)	RNP (*n* = 22)	CNP (*n* = 8)	
	Mean ± SD	Mean ± SD	Mean ± SD	
Maximal strength (MVC)				
Flexion MVC (kg)	20.2 ± 9.7	14.6 ± 6.4	15.3 ± 3.1	0.17 ^1^
Extension MVC (kg)	29.6 ± 18.5	15.3 ± 4.4 *	21.6 ± 9.1	0.006 ^1^
Rate of perceived exertion (BORG scale: 6–20)				
Flexion Borg (6–20)	12.0 ± 3.1	15.0 ± 3.0 *	14.7 ± 1.7	0.01
Extension Borg (6–20)	8.9 ± 2.5	9.9 ± 2.5	10.4 ± 2.6	0.38 ^1^

SD: standard deviation; SD error: standard error (of the mean); CI: confidence intervals; MVC: maximal voluntary contraction. ^1^ Kruskal–Wallis ANOVA. * Post hoc significant difference from control group at *p* < 0.05.

**Table 6:** Selected predictor variables for response variable of number of days with pain.

	NDI at 6 Months	Number of Days with Pain
(Intercept)	8.65	4.68
NDI	0	0
TSK	0	0
EQ-VAS	0	0
EQ-5D	0	0
Previous number of pain episodes	0.68	0.57
Average of pain episodes	0	0
ROM in flexions and extension	0	0
ROM in rotations	0	0
NVP in flexions and extension	0	0
JPE	0	0
20% and 40 of CCF MVC force	0	0
60%, and 80% of CCF MVC force	0	0
CCF MVC	0	0
MVC during cervical flexion	−0.34	0
MVC during cervical extension	0	0

NDI: Neck Disability Index; TSK: Tampa Scale of Kinesiophobia; EQ-5D: European Quality of Life—5 Dimensions; EQ-VAS; self-rated health on a vertical visual analogue scale; RoM: range of motion; NVP: number of velocity peaks; JPE: joint position error; CCF MVC: maximum craniocervical flexion strength; MVC: maximal voluntary contraction.

**Table 7:** Results of multivariate regression analysis showing associations between baseline predictors and NDI at six months.

	β	SE	*t* Value	*p* Value	Low 95%CI	Upper 95% CI	Adjusted R^2^
(Intercept)	10.23	2.99	3.42	0.004	3.82	16.63	0.43
MVC flexion	−0.32	0.15	−2.21	0.04	−0.64	−0.01	
Previous number of pain episodes	0.54	0.21	2.56	0.02	0.09	0.99	

β: unstandardized coefficient; SE: standard error; CI: confidence interval; Adjusted R^2^: represents the variance in NDI (the outcome) as explained by the variables; MVC: maximum voluntary contraction. *n* = 19; 86% with complete cases

**Table 8:** Results of multivariate regression analysis showing associations between baseline predictors and number of days with pain (average of 12 months).

	β	SE	*t* Value	*p* Value	Low 95%CI	Upper 95% CI	Adjusted R^2^
(Intercept)	2.14	1.17	1.83	0.08	−0.33	4.61	0.25
Previous number of pain episodes	0.40	0.15	2.63	0.02	0.08	0.71	

β: unstandardized coefficient; SE: standard error; CI: confidence interval; Adjusted R^2^: represents the variance in number of days with pain (the outcome) as explained by the variable. *n* = 17; 77% with complete cases.

## Data Availability

Raw data that support the findings of this study are available from the corresponding author, upon reasonable request.

## Supplementary Materials

The following supporting information can be downloaded at: https://www.mdpi.com/article/10.3390/jcm11072042/s1, Figure S1: Boxplots of NDI, TSK, EQ-5D, and EQ VAS of all three groups. Results of Post hoc tests between groups are presented; Figure S2: Boxplots of cervical movement of all three groups. Results of Post hoc tests between groups are presented; Figure S3: Boxplots of neck proprioception of all three groups. Results of Post hoc tests between groups are presented; Figure S4: Boxplots of mean velocity of all three groups. Results of Post hoc tests between groups are presented; Figure S5: Boxplots of smoothness of movement of all three groups. Results of Post hoc tests between groups are presented; Figure S6: Boxplots of normalized EMG recorded from SCM during submaximal craniocervical flexion task. Results of Post hoc tests between groups are presented; Figure S7: Maximal neck strength in flexion (A) and extension (B). Borg’s scale was used to measure perceived fatigue during submaximal contraction at 20% MVC; Figure S8: line plot showing mean number of days with pain (outcome) over 12 months follow-up period; Figure S9: Results of the Least absolute shrinkage and selection operator involving all predictors with Neck Disability Index as an outcome at 6 months; Figure S10: Results of the Least absolute shrinkage and selection operator involving all predictors with days with pain over the 12-month follow-up period as an outcome. Table S1: STROBE Statement—Checklist of items that should be included in reports of cross-sectional studies.
