# Supplementary material for: Assessment of Neuromuscular and Psychological Function in People with Recurrent Neck Pain during a Period of Remission: Cross-Sectional and Longitudinal Analyses
Source: J Clin Med. 2022 Apr 6;11(7):2042. doi: 10.3390/jcm11072042 (PMC8999485; doi:10.3390/jcm11072042)
Supplement: Supplementary file 1 [file jcm-11-02042-s001.zip › jcm-1671843-supplementary.pdf]

Supplementary Materials

Boxplots for self-reported questionnaire

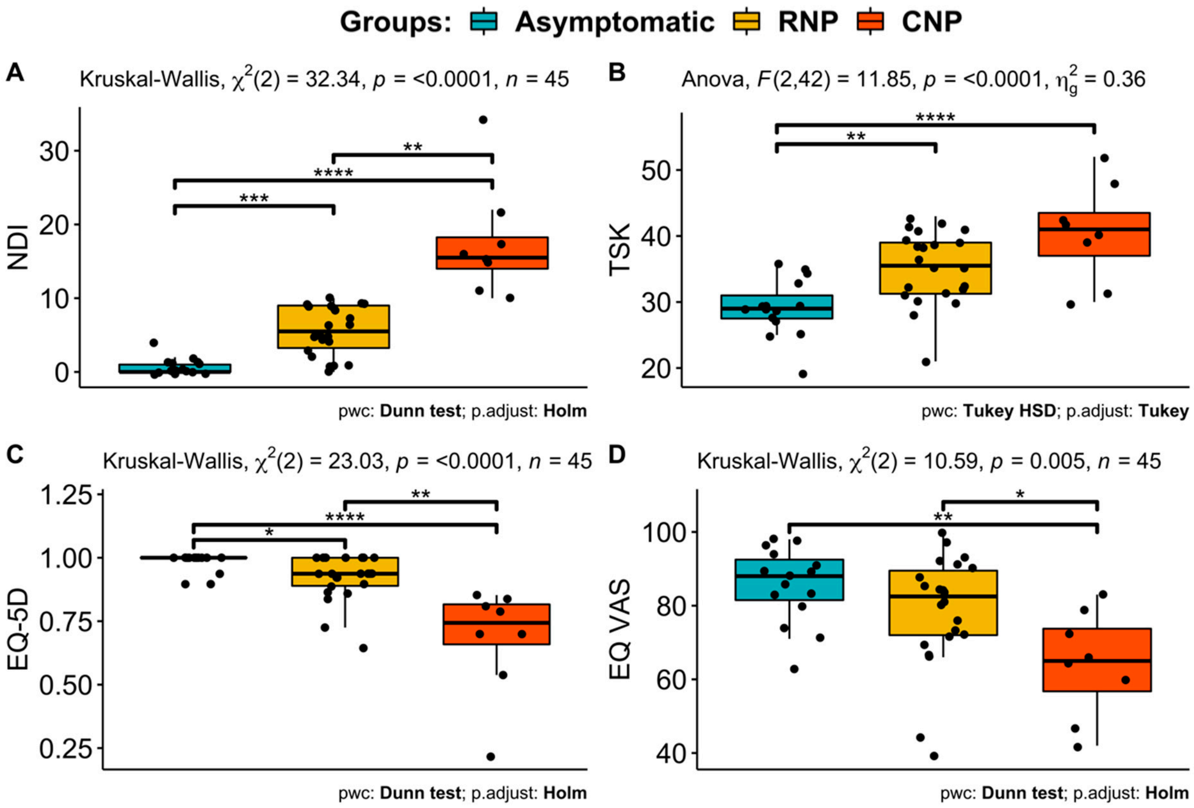

Figure S1: Boxplots of NDI, TSK, EQ-5D, and EQ VAS of all three groups. Results of Post hoc tests between groups are presented. *p<0.05, **p<0.01, *** p<0.001, **** p<0.0001
RNP: Recurrent Neck Pain; CNP: Chronic Neck Pain; NDI: Neck Disability Index; TSK: Tampa Scale of Kinesiophobia; EQ-5D: European Quality of life – 5 Dimensions; EQ-VAS; self-rated health on a vertical visual analogue scale; ; PWC: The post-hoc test used for the multiple pairwise comparisons; P.adjust: Method for calculating the adjusted p value.

Boxplots for cervical kinematics and proprioception

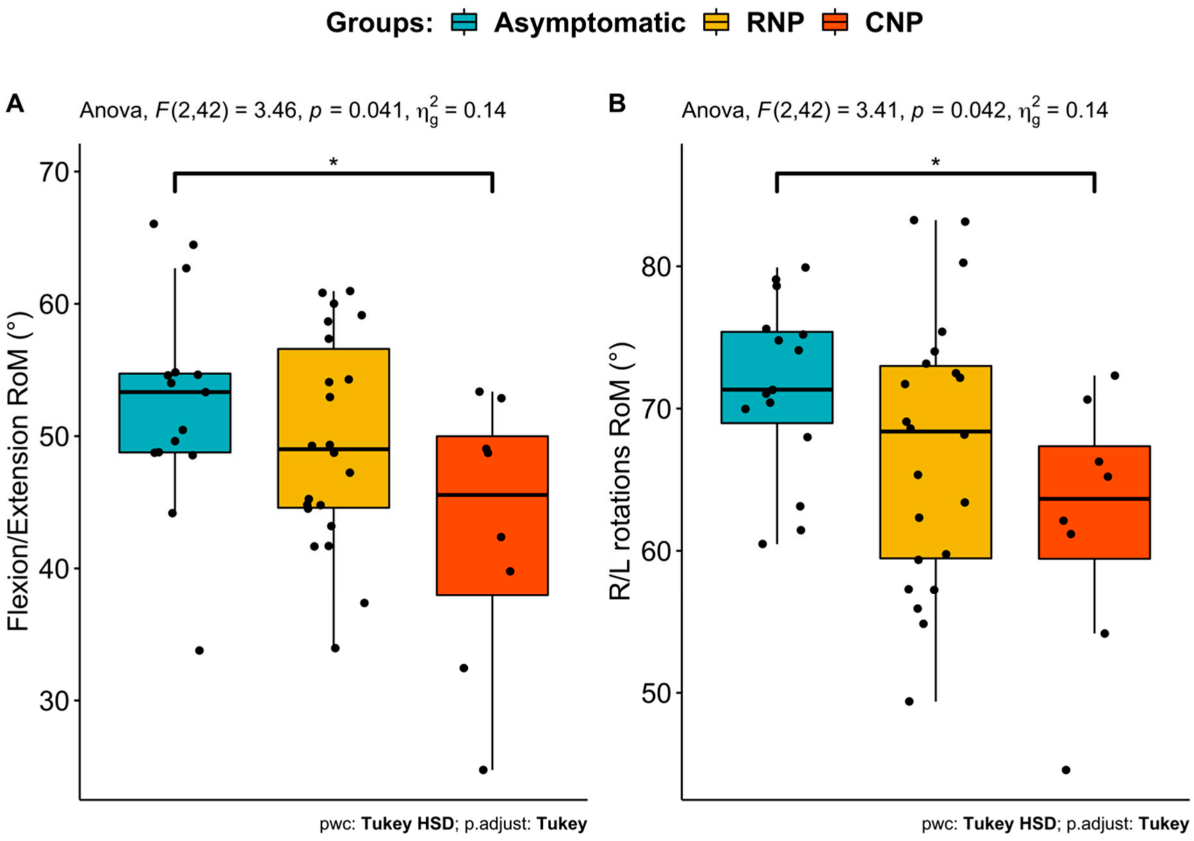

Figure S2: Boxplots of cervical movement of all three groups. Results of Post hoc tests between groups are presented; *p<0.05.
RNP: Recurrent Neck Pain; CNP: Chronic Neck Pain; RoM: Range of Motion; °: Degree; R/L: Right/Left; PWC: The post-hoc test used for the multiple pairwise comparisons; P.adjust: Method for calculating the adjusted p value.

Boxplots for joint position error

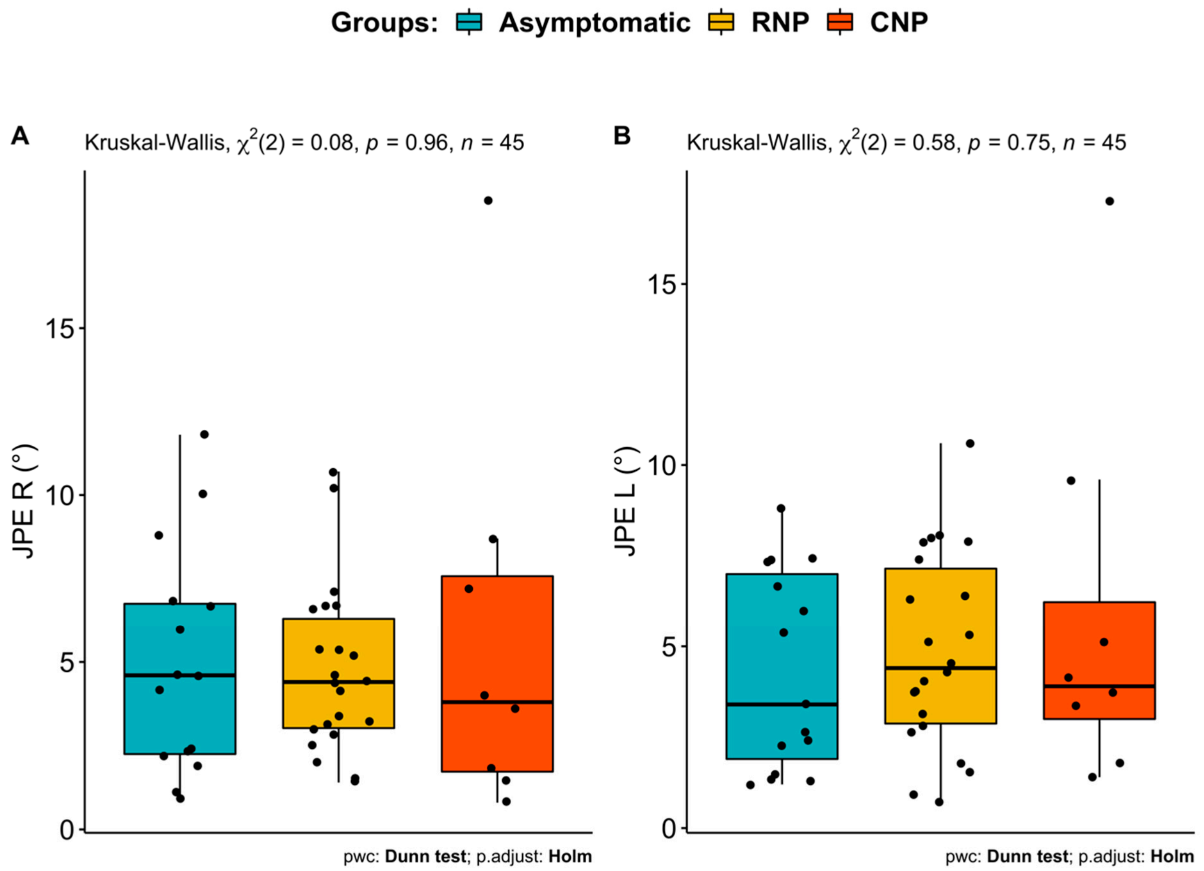

Figure S3: Boxplots of neck proprioception of all three groups. Results of Post hoc tests between groups are presented.
RNP: Recurrent Neck Pain; CNP: Chronic Neck Pain; JPE: Joint Position Error; °: Degree; PWC: The post-hoc test used for the multiple pairwise comparisons; P.adjust: Method for calculating the adjusted p value.

Boxplots for mean velocity in all directions

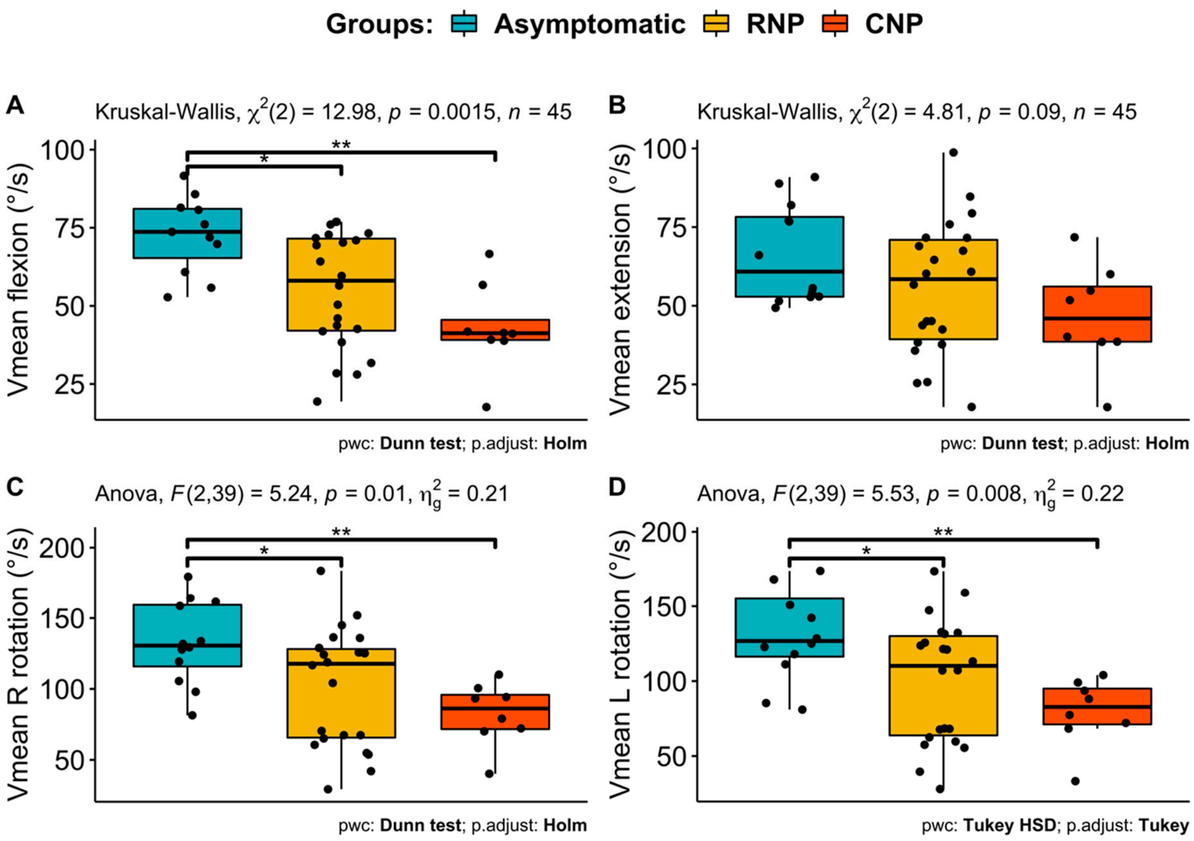

Figure S4: Boxplots of mean velocity of all three groups. Results of Post hoc tests between groups are presented; *p<0.05; **p<0.01.
RNP: Recurrent Neck Pain; CNP: Chronic Neck Pain; Vmean: Mean Velocity; °: Degree; S: Seconds; R: Right; L: Left; PWC: The post-hoc test used for the multiple pairwise comparisons; P.adjust: Method for calculating the adjusted p value.

Boxplots for number of velocity peaks in all directions

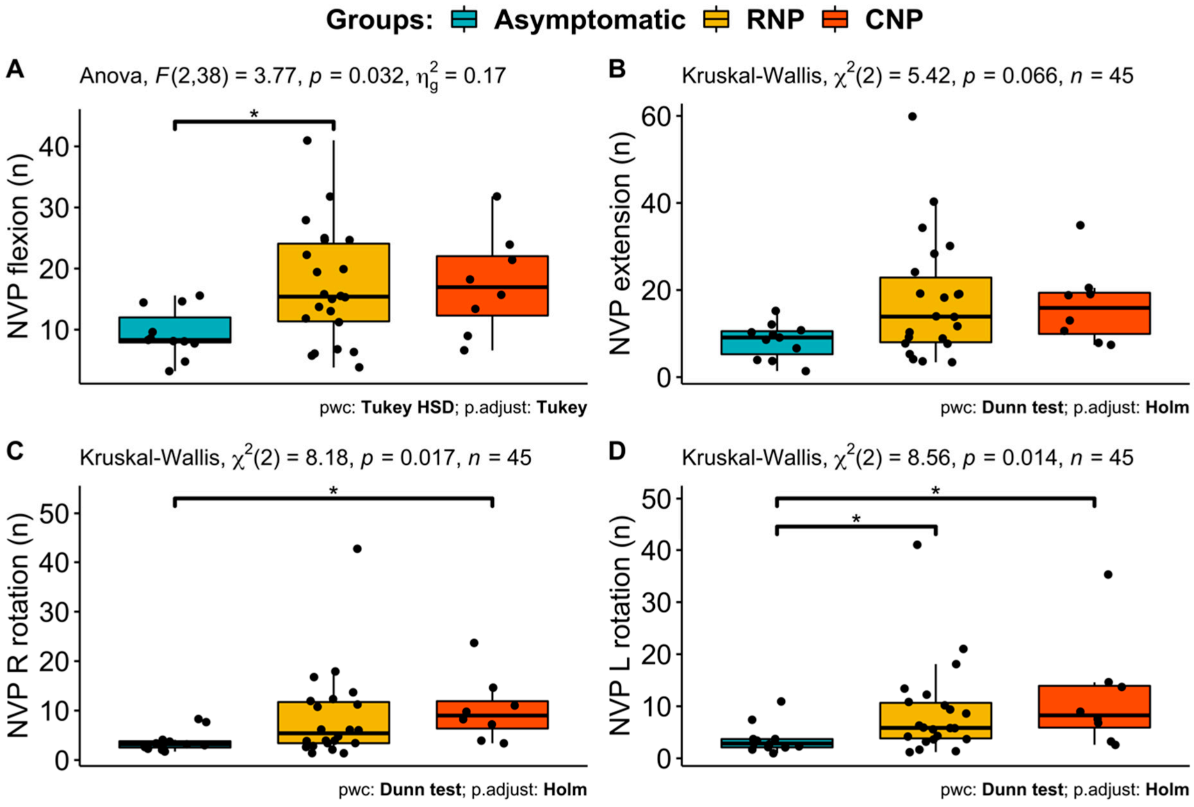

Figure S5: Boxplots of smoothness of movement of all three groups. Results of Post hoc tests between groups are presented. *p<0.05.
RNP: Recurrent Neck Pain; CNP: Chronic Neck Pain; NVP: Number of Velocity Peaks; n: Number; R: Right; L: Left; PWC: The post-hoc test used for the multiple pairwise comparisons; P.adjust: Method for calculating the adjusted p value.

Boxplots for EMG amplitude assessed during submaximal CCF contractions

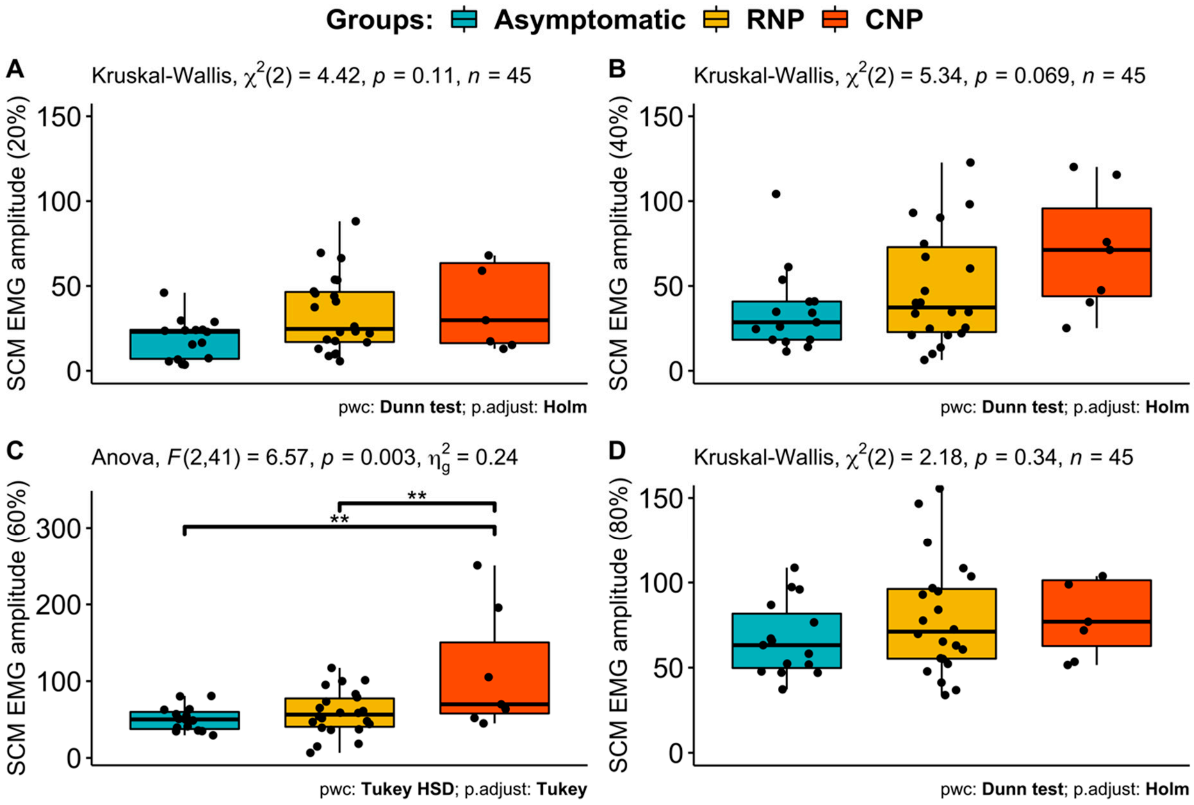

Figure S6: Boxplots of normalized EMG recorded from SCM during submaximal craniocervical flexion task. Results of Post hoc tests between groups are presented. **p<0.01.
RNP: Recurrent Neck Pain; CNP: Chronic Neck Pain; SCM: Sternocleidomastoid Muscles; EMG: Electromyography; PWC: The post-hoc test used for the multiple pairwise comparisons; P.adjust: Method for calculating the adjusted p value.

Boxplots for maximal neck strength and perceived fatigue

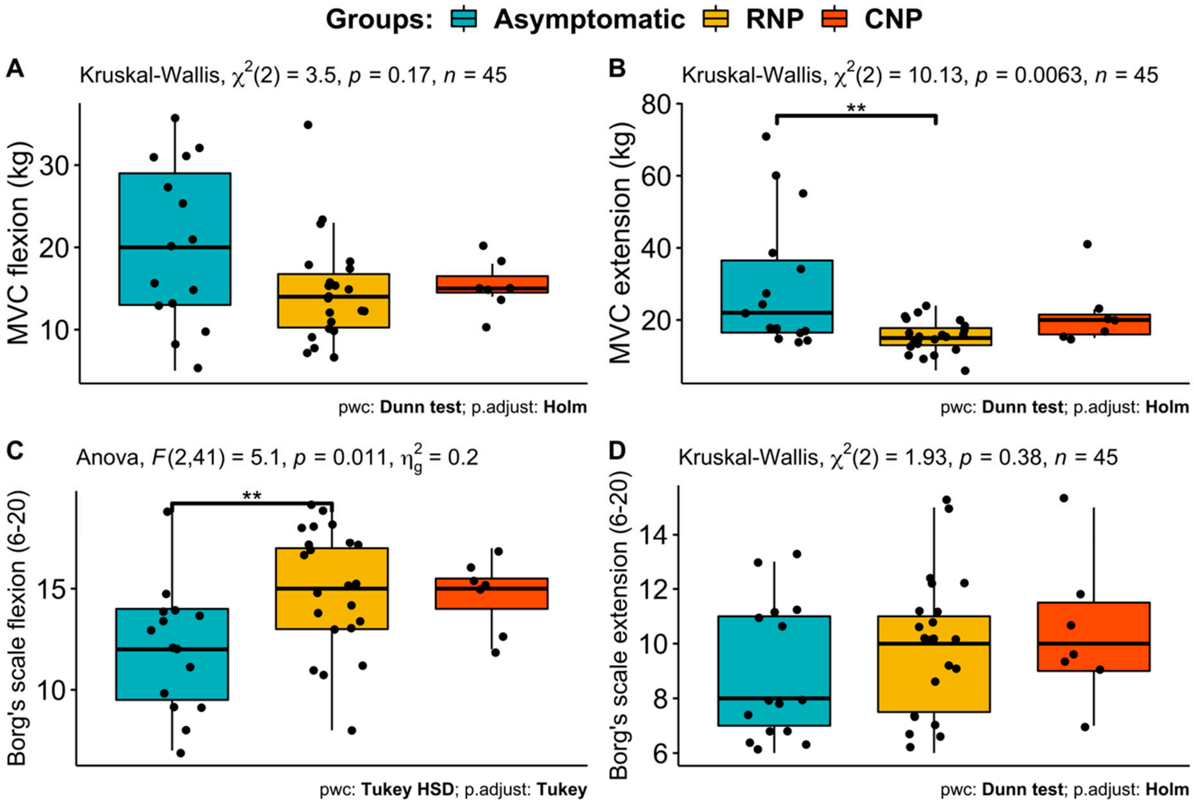

Figure S7: Maximal neck strength in flexion (A) and extension (B). Borg's scale was used to measure perceived fatigue during submaximal contraction at 20% MVC. **p<0.01.

RNP: Recurrent Neck Pain; CNP: Chronic Neck Pain; MVC: Maximum Voluntary Contraction: kg: Kilogram; PWC: The post-hoc test used for the multiple pairwise comparisons; P.adjust: Method for calculating the adjusted p value.

Mean values of number of days with pain over 12 months follow-up period

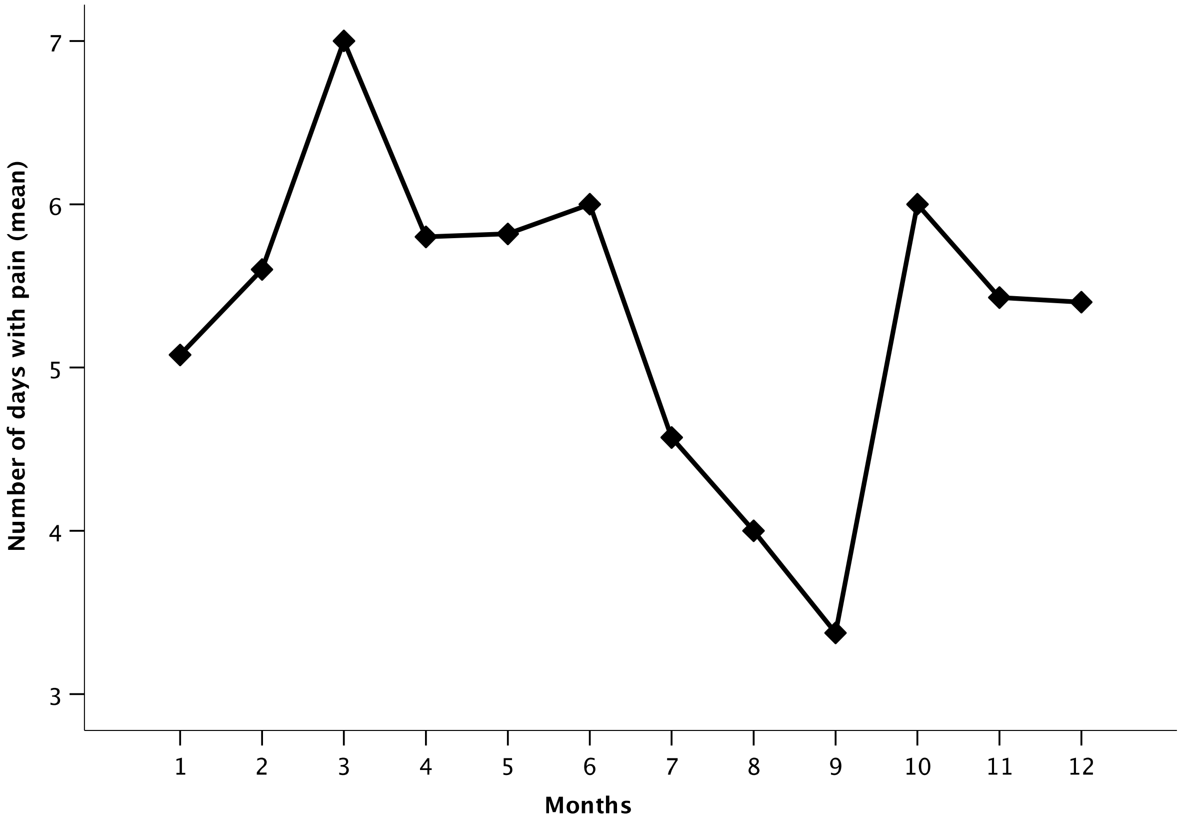

Figure S8: line plot showing mean number of days with pain (outcome) over 12 months follow-up period.

Graph for coefficients paths of LASSO regression (outcome: NDI at 6 months)

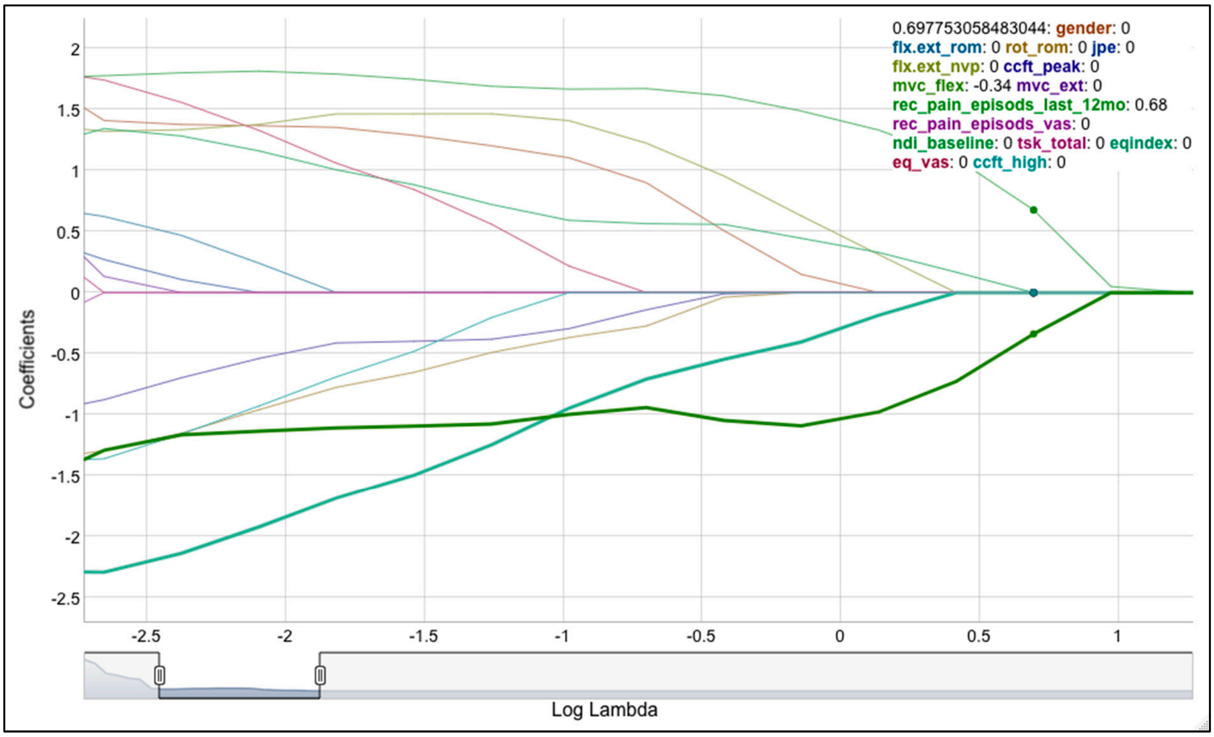

Figure S9: Results of the Least absolute shrinkage and selection operator involving all predictors with Neck Disability Index as an outcome at 6 months.
Legend on the right upper corner showing the included predictors in LASSO where all of them were shrined to Zero, except for mvc_flex = -0.34 and rec_pain_episodes_last_12mo = 0.68 .
mvc_flex: MVC in flexion ; rec_pain_episodes_last_12mo: Previous number of pain episodes in the last 12 months.

Graph for coefficients paths of LASSO regression ((outcome: number of days with pain over the 12-month period)

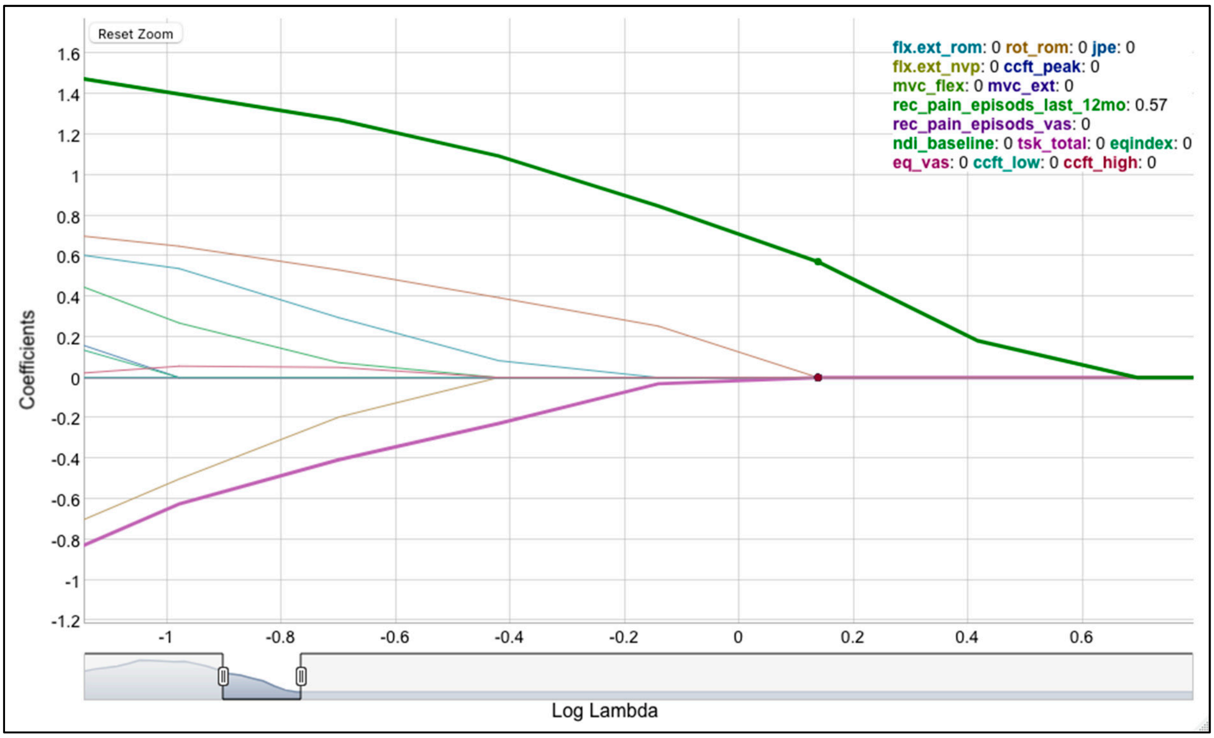

Figure S10: Results of the Least absolute shrinkage and selection operator involving all predictors with days with pain over the 12-month follow-up period as an outcome. Legend on the right upper corner showing the included predictors in LASSO where all of them were shrined to Zero, except for rec_pain_episodes_last_12mo = 0.57 . rec_pain_episodes_last_12mo: Previous number of pain episodes in the last 12 months.

Table S1. STROBE Statement—Checklist of items that should be included in reports of ***cross-sectional studies***

	Item No	Recommendation	Page No
**Title and abstract**	1	(*a*) Indicate the study's design with a commonly used term in the title or the abstract	1
		(*b*) Provide in the abstract an informative and balanced summary of what was done and what was found	1
**Introduction**			
Background/rationale	2	Explain the scientific background and rationale for the investigation being reported	1-2
Objectives	3	State specific objectives, including any prespecified hypotheses	1
**Methods**			
Study design	4	Present key elements of study design early in the paper	1
Setting	5	Describe the setting, locations, and relevant dates, including periods of recruitment, exposure, follow-up, and data collection	3-5
Participants	6	(*a*) Give the eligibility criteria, and the sources and methods of selection of participants	3-4
Variables	7	Clearly define all outcomes, exposures, predictors, potential confounders, and effect modifiers. Give diagnostic criteria, if applicable	4,7-9
Data sources/ measurement	8*	For each variable of interest, give sources of data and details of methods of assessment (measurement). Describe comparability of assessment methods if there is more than one group	5-7
Bias	9	Describe any efforts to address potential sources of bias	NA
Study size	10	Explain how the study size was arrived at	9
Quantitative variables	11	Explain how quantitative variables were handled in the analyses. If applicable, describe which groupings were chosen and why	9
Statistical methods	12	(*a*) Describe all statistical methods, including those used to control for confounding	9
		(*b*) Describe any methods used to examine subgroups and interactions	NA
		(*c*) Explain how missing data were addressed	10
		(*d*) If applicable, describe analytical methods taking account of sampling strategy	NA
		(*e*) Describe any sensitivity analyses	NA
**Results**			
Participants	13*	(a) Report numbers of individuals at each stage of study—eg numbers potentially eligible, examined for eligibility, confirmed eligible, included in the study, completing follow-up, and analysed	10, 13
		(b) Give reasons for non-participation at each stage	13
		(c) Consider use of a flow diagram	3
Descriptive data	14*	(a) Give characteristics of study participants (eg demographic, clinical, social) and information on exposures and potential confounders	10
		(b) Indicate number of participants with missing data for each variable of interest	13
Outcome data	15*	Report numbers of outcome events or summary measures	13
Main results	16	(*a*) Give unadjusted estimates and, if applicable, confounder-adjusted estimates and their precision (eg, 95% confidence interval). Make clear which confounders were adjusted for and why they were included	NA

		(*b*) Report category boundaries when continuous variables were categorized	NA
		(*c*) If relevant, consider translating estimates of relative risk into absolute risk for a meaningful time period	NA
Other analyses	17	Report other analyses done—eg analyses of subgroups and interactions, and sensitivity analyses	NA
**Discussion**			
Key results	18	Summarise key results with reference to study objectives	15
Limitations	19	Discuss limitations of the study, taking into account sources of potential bias or imprecision. Discuss both direction and magnitude of any potential bias	18-19
Interpretation	20	Give a cautious overall interpretation of results considering objectives, limitations, multiplicity of analyses, results from similar studies, and other relevant evidence	15-18
Generalisability	21	Discuss the generalisability (external validity) of the study results	18-19
**Other information**			
Funding	22	Give the source of funding and the role of the funders for the present study and, if applicable, for the original study on which the present article is based	19

*Give information separately for exposed and unexposed groups.

Note: An Explanation and Elaboration article discusses each checklist item and gives methodological background and published examples of transparent reporting. The STROBE checklist is best used in conjunction with this article (freely available on the Web sites of PLoS Medicine at http://www.plosmedicine.org/, Annals of Internal Medicine at http://www.annals.org/, and Epidemiology at http://www.epidem.com/). Information on the STROBE Initiative is available at www.strobe-statement.org.
